# Pott’s Disease

**Published:** 1879-01-20

**Authors:** Lewis A. Sayre

**Affiliations:** New York


### *POTT'S DISEASE.*

BY LEWIS A. SAYRE, M.D., OF NEW YORK.

Gentlemen, I shall not attempt to give you anything like a full lecture on this subject, but propose to make a few practical remarks and application of the treatment in the cases before us, so that you can understand the main points as well as the details of the treatment, and thus be enabled to do your patients as much good as I or any one else can do.

I shall speak first of Pott's disease; now Pott's disease and lateral curvature are both deformities, but one is a deformity only, while the other (Pott's disease) is a disease.

We find this to be the result of inflammation and absorption of the bodies of the vertebræ. The misfortune is that no deformity is observed until this condition has ensued. Could we find this out in time, as we should do by correctly interpreting the symptoms presented, the disease could be arrested, and the patient cured without deformity. I believe the direct or exciting cause of Pott's disease to be traumatic, and in saying so, I do not desire to be understood as not allowing scrofula and other hereditary forms of transmitted evils *to predispose* to it when there is an *exciting cause*, but I do not believe it occurs except from traumatic origin. And many have done me the injustice to say that I do not credit such evils as impoverished blood caused from scrofula, phthisis, syphilis, etc., as conducive to the disease, because I deny that they produce it independent of some exciting cause. Some injury is necessary to develop the disease, even in the depraved constitution.

It may be a fall across the hearth-rug, a gentle tip, or some slight trouble which would suffice to develop the disease in the feeble constitution, and from constant irritation cause trouble at the distal end of the nerve, and hence Pott's disease from remote injury.

The majority of cases occur in robust, healthy children, because they do not *guard themselves against injuries* like the weakly, ill-nourished child. The healthy child goes romping and tumbling about, and gets an injury which finally results in Pott's disease, while the child predisposed to it is careful, and goes along and misses it oftentimes, because its bad health keeps it from exposure to violent exercise and accidents consequent upon such a life.

You will observe the young one afflicted with this disease endeavors to put on a natural splint by keeping the muscles of the body rigid and the back straight, and thus getting the relief which is only to be obtained in this way; if he stops, it is with the whole body; if he jumps, it is to alight upon the toes, and keep the vertebræ from a jar.

The treatment is *rest! rest!! rest!!!* to the part affected. Formerly this was obtained by keeping the patient upon the back for a long, long time; they may occasionally get well by this plan, but oftener they die from a worse condition of the general health, which often follows this rigid confinement. Rest, to be successful, even in the horizontal position, must be combined with extension.

The pressure from reflex muscular contraction will cause absorption, if allowed, and will leave the patient deformed. Now extension, and the plaster jacket, gives the diseased part rest, by removing the pressure; it gives extension and support, and allows the patient to walk around with comfort, thus receiving the advantage of healthful exercise while undergoing the necessary treatment.

To apply the plaster jacket, you must have first a good-fitting shirt, such as I show you here; it should be fastened with tapes under the perineum, so as to keep it from wrinkling; a pad of cotton, "the dinner pad," should be put over the abdomen, under the shirt. Now, under the apparatus, *suspend the patient with great care; never hurt a patient.*

The point of extension is "when they feel right." This lifts the diseased vertebræ off of one another, and straightens the spine.

The patient is now ready for the plaster bandage. The roller is made of crinoline, or crossbarred muslin; rub the plaster of Paris into it, and roll up ligthly. Now drop the rollers into the water, deeply enough to cover them over *endwise;* this drives out all gas. Now bind the roller, commencing just above the hips, so that the pelvis supports the body; apply smoothly and evenly some two or three times, and smoothe the wrinkles out as you proceed with the hand.

You must make the shirt fit like the skin, have equal and uniform pressure, and I defy you to have a slough. Now we take this child down, remove the pad from over the abdomen, this will leave room for a full meal. I take my hand and press the jacket down in the groins, thus making it fit the child everywhere; lay him flat down on his back until the plaster sets; I then turn him loose, and he can go on all right.

There is one great advantage in using plaster of Paris; it is porous, and you can breathe through it, so that the child can perspire, the air can reach his skin; if we were to varnish this child we would kill him.

To find whether a case is fixed or anchylosed, and cannot be straightened by extension, I take this malleable piece of soft metal strip, mould it along the spinal curve, take this curve on paper, put the patient under the extending apparatus; draw him up; let him swing long enough to overcome the muscles; take the curve again, and if it is the same, the case is irremediable and permanent, and should be let alone; if there is a new curve, you have a case for treatment.

NOTE.—The results of the cases which were treated by Dr. Sayre before the Medical Society were highly gratifying, and very striking; for example, a poor, weakly child, unable to get along at all, in a few minutes after Dr. Sayre had given him a *new back*, was running around, greatly to his own delight and his parent's joy.

We have only given Dr. Sayre's remarks on Pott's disease. We have made no mention of the head rest, or "jury mast," for we refer our readers to Dr. Sayre's book for a description of all apparatus used, as well as his treatment, and all other information connected with this trouble. No physician can afford to be without the work, and whoever once sees the glorious results of the treatment introduced by this great surgeon, will ever regard him as one of the great benefactors of the human race.—*Southern Clinic.*

